# Mechanochemical Preparation of Pd(II) and Pt(II) Composites with Carbonaceous Materials and Their Application in the Suzuki-Miyaura Reaction at Several Energy Inputs

**DOI:** 10.3390/molecules25122951

**Published:** 2020-06-26

**Authors:** Mohamed M.A. Soliman, Andreia F. Peixoto, Ana P.C. Ribeiro, Maximilian N. Kopylovich, Elisabete C.B.A. Alegria, Armando J.L. Pombeiro

**Affiliations:** 1 Centro de Química Estrutural, Complexo I, Instituto Superior Técnico, Universidade de Lisboa, 1049-001 Lisboa, Portugal; mohamed.soliman@tecnico.ulisboa.pt (M.M.A.S.); apribeiro@tecnico.ulisboa.pt (A.P.C.R.); pombeiro@tecnico.ulisboa.pt (A.J.L.P.); 2 Área Departamental de Engenharia Química, ISEL, Instituto Politécnico de Lisboa, 1959-007 Lisboa, Portugal; 3 REQUIMTE/LAQV, Departamento de Química e Bioquímica, Faculdade de Ciências, Universidade do Porto, Rua do Campo Alegre s/n, 4169-007 Porto, Portugal; andreia.peixoto@fc.up.pt

**Keywords:** mechanochemical, liquid-assisted grinding, Suzuki-Miyaura reaction, cross-coupling, Palladium, Platinum, composites

## Abstract

Pd(II) and Pt(II) composites with activated carbon (AC), graphene oxide, and multiwalled carbon nanotubes were prepared by ball milling and used as catalysts for the Suzuki-Miyaura reaction, under several energy inputs (mechanical grinding, conventional heating, and microwave irradiation). The catalytic composites were characterized by ICP-MS, BET, XPS analyses, TEM, and SEM. The average particle size of the prepared composites was estimated to be in the range of 6–30 nm, while the loadings of Pd and Pt did not significantly affect the surface area of the AC support due to the tendency to agglomerate as observed by the TEM analysis. The Pd/AC composites exhibit high mechanochemical catalytic activity in cross-coupling of bromobenzene and phenylboronic acid with molar yields up to 80% with TON and TOF of 222 and 444 h^−1^, respectively, achieved with Pd(4.7 wt%)-AC catalyst under the liquid assisted grinding for 0.5 h at ambient conditions, using cyclohexene as an additive.

## 1. Introduction

The Suzuki-Miyaura (SM) cross-coupling of aryl halides with aryl boronic acids to form biaryls has become an exceptionally powerful tool in organic synthesis [1,2,3,4]. The notable expansion of this reaction is based on its applicability to a wide range of substrates with various functional groups and availability of the boronic acid starting materials. Generally, this chemical transformation needs a transition metal catalyst to proceed at a synthetically useful rate, and catalysts based on Pd dominate the scene [1,2,3,4]. The homogeneous catalytic conditions were the first choice of chemists because of high activity and selectivity; however, difficulties in the catalyst recovery and product purification are obvious drawbacks even at the laboratory scale. Moreover, catalysts that are active but difficult to recycle/recover from reaction mixture are generally not favored in the chemical industry. Hence, the recovery and reusability of catalysts is critically important for a sustainable development of the SM cross-coupling processes. These issues can be overcome by e.g., the catalyst heterogenization on solid supports. However, in many cases the heterogenization decreases the overall catalytic efficiency, being also expensive and difficult to achieve on industrial scale. Therefore, elaboration of simple, scalable, eco-friendly and fast technics to prepare highly active and reusable dispersed catalysts for the SM reaction constitutes a significant challenge [5,6,7], and mechanochemical methods can eventually provide an effective solution [8,9,10,11,12].

The mechanochemical preparative technics involve chemical and physico-chemical transformations of substances produced by the effect of mechanical energy. These include influencing reactivity of solids by the presence of solid-state defects, interphase interactions and relaxations, which can enable time-convenient one-step syntheses of new nanomaterials with the desired properties in a reproducible way with high yields and under simple and easy operating conditions [8,9,10,11,12]. Due to these and other phenomena (e.g., formation of “hot spots” or mechanically stimulated phase transformations) [8], many mechanochemical processes take place under environmentally friendly and essentially waste-free conditions. Another advantage of the mechanochemical preparative technics involves their easy scaling up with the industrial milling equipment already available [8].

As it was mentioned above, Pd(II) salts or complexes are usually employed as catalytic agents for cross-couplings and related reactions. Such compounds are then reduced in situ to Pd(0) species which enter the catalytic cycle. The catalytic cycle involves the rate-determining activation of the aryl halide by oxidative addition, transmetalation and reductive elimination [1,2,3,4,5,6,7]. In each of these steps an effective charge transport from the metal nanoparticles to the π system of the aryl halides or boronic acids is important, and hence physicochemical properties of the support are relevant for the successful metal heterogenization, dispersion, charge transport, and, as result, the overall catalytic activity.

The delocalized system of π electrons of carbonaceous materials is beneficial for π-π interactions with aromatic groups of substrates and for constructing electron-enriched vacancy defects which facilitate redox processes [9,13,14,15,16]. Due to these reasons as well as their high chemical stability, mechanical resistance, large surface area, tunable electronic, and physicochemical features, the carbon-based materials are broadly used as catalysts and catalyst supports in many industrially important processes [17,18,19,20,21], while mechanochemical methods have been applied for their preparation and functionalization [9,10,11,12,22,23,24]. Moreover, the use of supported Pd may prevent its aggregation upon reduction with the respective drop in activity [1,2,3,4,5,6,7]. Among different carbon materials, activated carbon (AC) is the most used and studied support for Pd catalysts and in the C-C coupling chemistry due to its low price and abundance, unique chemical and mechanical properties [20,21,25,26,27,28,29]. Thus, carbon is a high tensile material, able to sustain the amount of energy produced in ball milling, with the advantage of not inhibiting the SM reaction. Accordingly, in this study we intend to develop this line further and to prepare new composites composed from palladium salts and activated carbon as a support and component of catalytic system. For comparative purposes, platinum salts will be also tried as the main catalytic species and graphene oxide (GO) and multiwalled carbon nanotubes (MWCNT) as supports.

**Scheme 1:**
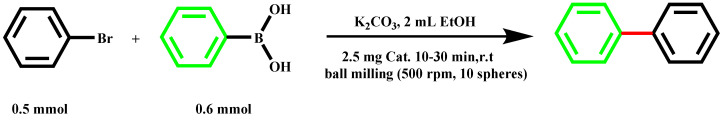
Suzuki-Miyaura reaction of bromobenzene and phenylboronic acid (typical reaction conditions)**.**

From another prospective, not only the preparation of catalysts can be achieved by mechanochemical procedures, but also the catalytic reactions themselves [10,12]. The application of mechanical energy to promote C-C coupling is a hot topic nowadays, and several prominent papers have been published highlighting different aspects of this field [30,31,32,33,34]. The advantages of the mechanochemical treatment include decreased reaction times, simplified work-up procedures, omission or minimization of solvents and better energy balance in comparison with the conventional procedures. According to this view, in this study we intend to prepare “(metal salt)-(carbon material)” composites (where metal is Pd or Pt and carbon material is AC, GO, or MWCNT) by simple mechanochemical procedure and apply the prepared composites as catalysts in the mechanically activated SM model reaction, namely coupling of phenylboronic acid and bromobenzene as the aryl halide partner (Scheme 1). For comparative purposes, other energy inputs, such as the conventional heating in oil bath with magnetic stirring and microwave (MW) irradiation, were also studied.

## 2. Results and Discussion

### 2.1. Preparation of Pd and Pt Catalysts

Pd(II) and Pt(II) composites were prepared by ball milling using their respective Pd(CH_3_COO)_2_ and K_2_PtCl_4_ salts as Pd and Pt sources, respectively, and commercially available AC as support (see Section 3.1., Table 1 and Electronic Supporting Material for details). The approximate theoretical metal-to-carbon ratio was 4.7 and 8.5 wt%, while the actual metal content was determined by Inductively Coupled Plasma-Mass Spectroscopy (ICP-MS, Table 2). The prepared catalysts will hereinafter be denoted as AC-Pd1 and AC-Pt1 for the theoretical metal content of ca. 4.7 wt%, and AC-Pd2 and AC-Pt2 for the respective contents of ca. 8.5 wt%. For comparative purposes, palladium composites with GO and MWCNT were similarly prepared by grinding in ball mill. As it was indicated above, the use of the carbon-based material aims to improve the catalyst activity and stability in the reaction medium, allowing its recovery and reuse [2,3,4,5,6,7,17,18,19,20,21,25,26,27,28,29].

### 2.2. Characterization of the Pd/AC and Pt/AC Composites

To define the surface morphology of the prepared composites, a set of characterization techniques [ICP-MS, Scanning Electron Microscopy (SEM) and Energy Dispersive X-ray Spectroscopy (EDX), Transmission Electron Microscopy (TEM), X-ray Photoelectron Microscopy (XPS) and Brunauer-Emmett-Teller Surface Area Analysis (BET)] has been undertaken.

ICP-MS analysis of the supported Pd and Pt catalysts was carried out to evaluate the actual loading amounts of Pd and Pt on AC. As can be seen from Table 2, the actual loading amounts of Pd and Pt on AC are 4.13 wt% (for AC-Pd1), 7.63% (for AC-Pd2), 4.47% (for AC-Pt1), and 7.28% (for AC-Pt2), respectively, giving more than 80% of the full deposition.

**Figure 1:**
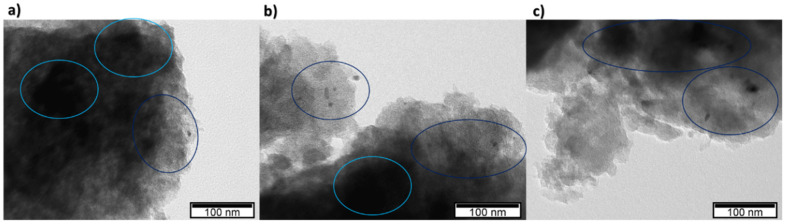
TEM images of AC-Pd1 (**a**), AC-Pd2 (**b**) and AC-Pt2 (**c**).

**Figure 2:**
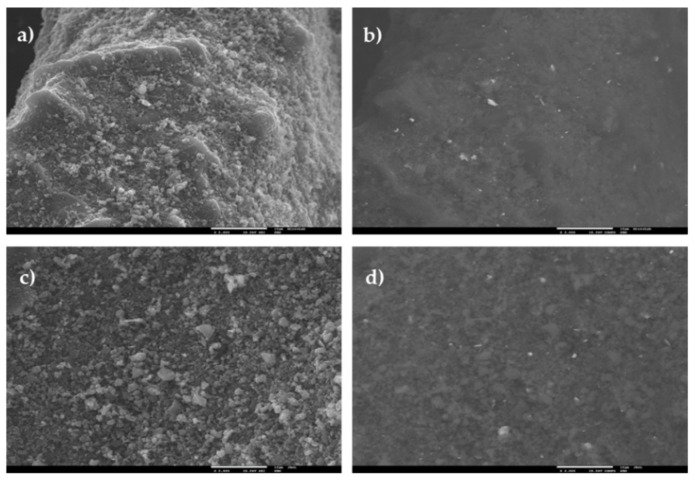
SEM images of AC-Pd1 (**a**,**b**), AC-Pd2 (**c**,**d**), 10 µm scale (**a**,**c**) and 2000× magnification (**b**,**d**).

**Figure 3:**
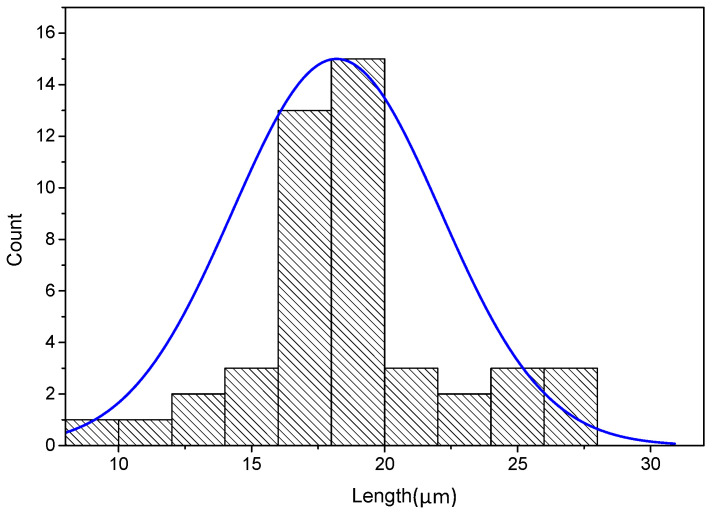
Particle size distribution in the AC-Pd2 sample.

The performed TEM (Figure 1) and SEM (Figure 2) confirmed the presence of Pd and Pt particles supported on the AC surface. The produced dispersed materials consist of non-uniform crystallites with tendency to agglomerate (ovals in Figure 1); this creates some difficulties to define the particle size and distribution. Nevertheless, the evaluation of the particle sizes and/or agglomerates present in the prepared materials can be performed by the digital microscopy Motic plus image software, giving average particle size around 18 nm for the AC-Pd2 dispersed nanomaterial (Figure 3).

The SEM images for the AC-Pd1 (Figure 2a) and AC-Pd2 (Figure 2c) composites demonstrate micro (below 1 µm) irregularities. In the back-scatter mode, the metal can be differentiated from support; elements of greater atomic mass (e.g., Pd) appear brighter in a back scattered electron image. Both images were collected at magnification 2000× (Figure 2b,d for AC-Pd1 and AC-Pd2, respectively) and, as expected, the visible amount of dispersed Pd is low, what is in accordance with low Pd loadings. The EDX analysis was performed for AC-Pd1 (Figure S1a) and AC-Pd2 (Figure S1b) composites to determine the elemental composition and to confirm that Pd is the only hetero element. The EDX analysis allowed to conclude that possible contamination due to the multiple collisions between medium, reactor, and grinding balls, detected in some mechanochemical reactions [35,36,37,38,39], was not observed.

**Figure 4:**
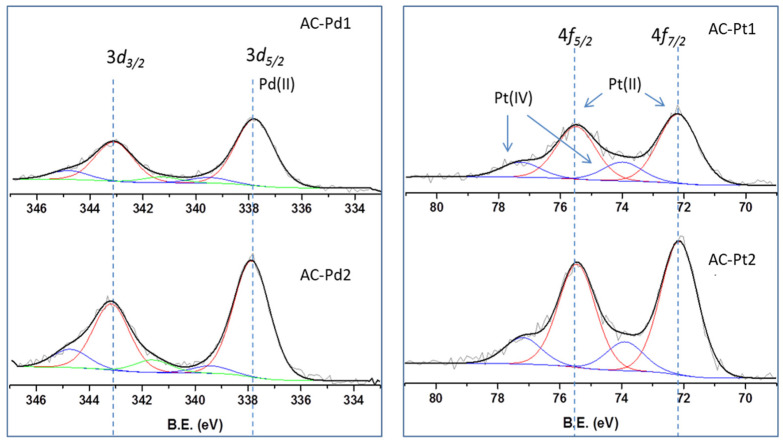
XPS profiles of the catalysts in the Pd 3d and Pt 4f regions.

The qualitative and quantitative surface characterization of the Pd/AC and Pt/AC composites were also performed by XPS (Figure 4, Figures S2 and S3 and Table S1). No significant changes were observed in the high-resolution spectra of the catalysts prepared with different content of Pt or Pd precursors. High resolution Pd 3d region spectra presents a doublet corresponding to Pd 3d_5/2_ and Pd 3d_3/2_ at 337.8 and 343.1 eV suggesting the predominant presence of Pd(II) species in the surface of activated carbon (>80%) [40]. The presence of Pd(0) was not detected on the surface, although previously being identified by other authors in similar systems [41]. The small peaks observed at 340.0, 341.7 and 345.2 eV could be related to parental species from the Pd precursor [42]. The deconvolution of high-resolution spectra Pt 4f region (Figure 4) revealed two bands, each comprised of a pair of doublets: the Pt 4f_7/2_ band at low binding energy 72.2 eV and Pt 4f_5/2_ band at 75.5 eV assigned to Pt(II). The other two peaks at higher BEs 74.2 (Pt4f_7/2_) and 77.5 eV (Pt4f_5/2_) correspond to Pt(IV) [43]. The presence of divalent platinum in this catalyst could be due to decomposition of the platinum precursor during ball milling [44]. XPS survey analysis also revealed the presence of chlorine species on the carbon surface (Figure S2) showing an incomplete decomposition of the platinum precursor under the used experimental conditions.

In the carbon spectra a small peak at 290 eV was observed revealing the presence of carboxyl groups, in addition to other typical peaks including the main peak of carbon at 284.6 eV (Figure S3). Additionally, the high resolution O1s region spectra of the composites (Figure S3) give additional information about surface oxygen groups, including the peaks at ~531.5 eV attributed to C-O/C=O and M-O functional groups and different peaks at 534.2 eV for Pd/AC and at 533.3 eV for Pt/AC composites attributed to the presence of carboxylic acid and anhydride functional groups, respectively. The contribution of chemisorbed water located at 536 eV was observed in the Pd/AC samples. The ratio of total oxygen to total carbon, O/C (Table S1,) relates to the surface oxidation [45]. An increase in the oxygen content for the Pd/AC composites was observed in comparison to the pristine AC, probably as result of the carbon bonds cleavage during ball milling with the respective surface oxidation or due to a decomposition of the metal salt [41]. The XPS contents of Pd and Pt (Table S1) are lower to those obtained by ICP (Table 1) possibly due to the tendency of the Pd and Pt nanoparticles to agglomerate, as supported also by SEM and TEM (see above).

**Figure 5:**
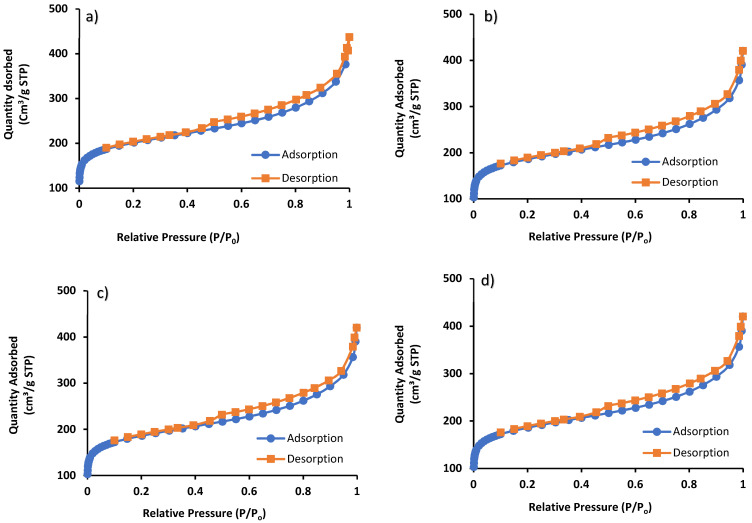
N_2_ adsorption-desorption isotherms of synthesized AC-Pd1 (**a**), AC-Pd2 (**b**), AC-Pt1 (**c**), and AC-Pt2 (**d**) composites.

The nitrogen adsorption-desorption isomers for AC (Figure S7), Pd/AC and Pt/AC composites (Figure 5) were determined at −196 °C. According to the IUPAC classification [46,47,48], a shape type (IV) isotherms and type IV (H4) hysteresis loops were observed in the relative pressure range from 0.4 to 1.0 indicating the presence of mesopores, the formation of asymmetric, slit-shaped mesopores, attributable to rapid gas evolution and open channels [46,47,48].

Table 3 compares the BET surface areas, pore volume and pore size of the commercial AC with those of the Pd/AC and Pt/AC supported catalysts. The surface area of the commercial AC was found to be 775 m^2^/g with a pore volume of 0.39 cm^3^/g and BET surface areas for the Pd/AC and Pt/AC composites arrange in the following order: AC-Pd1 (747 m^2^/g) > AC-Pt1 (722 m^2^/g) > AC-Pd2 (648 m^2^/g) > AC-Pt2 (584 m^2^/g). The surface area together with the type and percentage of supported metal correlates with the catalytic results (see below). It should also be noted that the micropores volume of the studied materials are close to the commercial AC value of 0.4 cm^3^/g, except of the AC-Pt2 composite which exhibits a lower value of 0.35 cm^3^/g. As can be seen, no drastic changes in the textural properties of the studied samples were observed, suggesting that the differences in the adsorption capacities are mainly due to their surface properties.

### 2.3. Catalytic Studies

In order to investigate the effects of various parameters on the SM reaction catalyzed by the prepared composites, a model cross-coupling of bromobenzene and phenylboronic acid has been selected. Additionally, the catalytic activity of the AC-Pd2 composite was compared with Pd catalysts supported on MWCNT, GO, and CaCO_3_ (Table 4). Several reaction parameters (solvent, base, reaction time) were also evaluated to maximize the yield of the coupling reaction. The commercial salts used for the preparation of composites were also tested with the same mass (2.5 mg) giving ca. 75% yield (TON 34) for Pd(OAc)_2_ catalyst vs. 49% (TON 136) for the AC-Pd2 material. As can be seen, 1.5 yield drop is well compensated by the saving in Pd content. The use of commercial K_2_PtCl_4_ resulted in only 3% yield, indicating the importance of the AC support in improving the catalytic activity.

**Figure 6:**
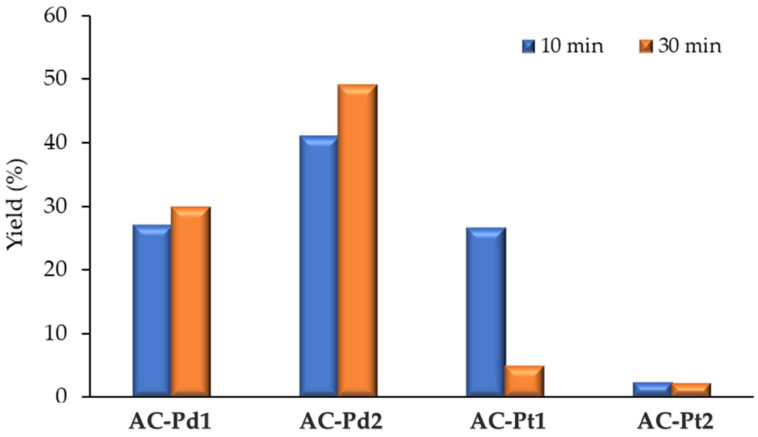
Cross-coupling Suzuki-Miyaura reaction of bromobenzene and phenylboronic, catalyzed by AC-Pd1, AC-Pd2, AC-Pt1, and AC-Pt2 composites. Reaction conditions: 2.5 mg catalyst, 0.6 mmol PhB(OH)_2_, 0.5 mmol PhBr, 1.0 mmol K_2_CO_3_, 2 mL EtOH, Ball mill: 10 milling spheres, 500 rpm, rotation interval (5 min) during 10 or 30 min.

Pt catalysts are not as common as the palladium ones for the SM reactions; however, application of the AC-Pt1 catalyst resulted in 26% yield (TON of 228 and TOF of 1.4 × 10^3^ h^−1^) of biphenyl after 10 min (Table 4, entry 16). However, after 30 min there was a drastic reduction in yield (Table 4, entry 17), probably due to the deactivation of the catalyst. Among the palladium-based materials, the use of the AC-Pd2 composite as a catalyst resulted in the highest yields up to 49% after 30 min and 70% after 180 min (Table 4, entries 6 and 7). The catalyst with higher metal content showed higher yield (30% for AC-Pd1 relatively to 49% for AC-Pd2 after 30 min, Table 4, entry 3); however, with some decrease in TONs (155 and 136, for AC-Pd1 and AC-Pd2, respectively, after 30 min). The nanocrystalline structure of the catalysts, the absence of contaminations and the large surface area are possible reasons for the considerable conversion exhibited by this catalyst (Figure 6).

Considering the above catalyst screening, the AC-Pd2 composite was selected for the more detailed catalytic studies. Thus, several inorganic bases, namely K_2_CO_3_, NaF, Na_2_CO_3_, KF, and Cs_2_CO_3_, were tested for this composite, and application of potassium carbonate resulted in the highest biphenyl yield of 49% (Table 4, entry 6, Figure S8). The use of Na_2_CO_3_, KF and Cs_2_CO_3_ gave lower and similar yields in the range of 31–35% (Table 4, entries 8–10), while NaF provided the lowest product yield of 2%. Although water is considered a green solvent, its use is not beneficial as well as application of dichloromethane (DCM), acetonitrile (ACN), and dimethyl sulfoxide (DMSO), while implementation of “green” ethanol significantly improves the yield (Table 4, entries 6 and 12–15, Figure S9).

To check if replacement of the AC support by the related carbon materials would influence the catalytic results, GO and MWCNTs were used as supports (Table 4, entries 20–24). As can be seen, the AC-Pd2 catalyst (yield of 49%, Table 4, entry 6) is slightly less active than the GO-Pd2 (yield of 53%, Table 4, entry 20) and CNT-Pd2 (yield of 50%, Table 4, entry 24) analogs, under the same reaction conditions (Figure S10), while the inorganic CaCO_3_ support provided a low yield of 12%, possibly due to inferior surface area.

The promoting effect of olefins [31,32], namely cyclohexene, cyclooctene and 1,5-cyclooctadiene (1,5-COD), was also explored for the so-named liquid-assisted grinding (LAG) process with the AC-Pd2 composite as a catalyst (Table 5). The presence of cyclooctene improved the yield to 59% with ethanol as a solvent (Table 5, entry 1), whereas the presence of cyclohexene and 1,5-COD dropped the yield to 19% and 21%, respectively (Table 5, entries 4 and 5). In solventless conditions, the yield increased from 59 to 80% and from 21 to 39% for the cyclooctene and 1,5-COD additives, respectively (Table 5, entries 1, 2, 5, and 6). Moreover, the addition of EtOH as an additive (0.4 µL/mg catalyst) resulted in 40% yield (Table 5, entry 7), while NaCl additive provided yield of only 28% (Table 5, entry 8).

**Figure 7:**
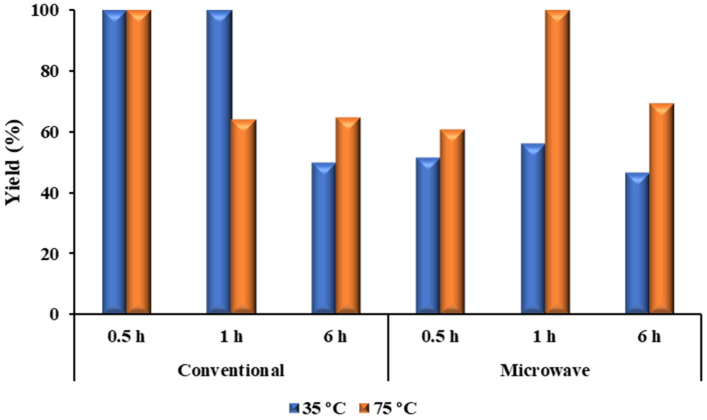
Conventional and microwave (MW) irradiation heating methods vs. time on the Suzuki-Miyaura (SM) reaction.

The effect of other than mechanical energy inputs on the catalytic output has been also studied (Table 6, Figure 7). As can be seen, in case of convectional heating (CH) for 1 h yield is higher at 35 °C in respect to 75 °C (Table 6, entry 2 vs. 5), probably due to the to the formation of Pd black with its agglomeration at higher temperatures. The CH after 30 min resulted in the complete conversion to biphenyl product, either at 75 or 35 °C, while MW irradiation has also a promoting effect since 60 min of reaction at 75 °C resulted in total conversion. However, the described above liquid assisted grinding is much more sustainable and environmentally friendly taking in consideration the energy necessary to produce and recover the used solvents.

## 3. Materials and Methods

The activated carbon (AC, DARCO^®^, −100 mesh particle size), palladium acetate Pd(OAc)_2_ 47.5% Pd, ethanol, toluene, ACN, DCM, cyclohexene, 1-cyclooctene, and 1,5-cyclooctadiene were purchased from Aldrich (Sigma-Aldrich, Munich, Germany). Potassium tetrachloroplatinate(II) K_2_PtCl_4_ was purchased from Alfa Aesar (Kandel, Germany), bromobenzene (99%) from Panreac (Panreac, Darmstadt, Germany) and phenylboronic acid (95%) from Fluka (Fluka, Buchs, Switzerland). None of the used commercial salts is significantly sensitive to air or moisture and all the samples were handled under ambient conditions, without additional purification.

### 3.1. Preparation of Catalysts

The Pd and Pt-based composites were synthesized as follows: 100 mg of support (AC, GO, or MWCNT) and 11 mg or 22 mg of commercially available palladium acetate Pd(OAc)_2_ or potassium tetrachloroplatinate(II) K_2_PtCl_4_ were added to a 250 mL stainless steel grinding bowl with 10 stainless steel balls of 10 mm diameter and milled in a Retsch PM100 planetary ball mill for 1 h at rotational speed 500 rpm, with rotational inversions every 5 min, at room temperature. All the composites were prepared in the absence of any added solvent (dry milling). All the samples were handled under ambient conditions, without additional purification of the commercial reagents.

### 3.2. Characterization of Catalysts

The microstructure analysis (SEM and EDX) of the catalysts was conducted with a scanning electron microscope JEOL 7001F (JEOL, Akishima, Tokyo, Japan) with Oxford light elements EDX detector and EBSD detector. The morphology of the composites was analyzed by TEM measurements and performed on a Hitachi 8100 microscope (Hitachi, Tokyo, Japan) with a ThermoNoran light elements EDX detector and digital image acquisition. ICP-MS was carried out by Microanalytical Service of the Instituto Superior Técnico. XPS was performed with a Kratos AXIS Ultra HAS (KRATOS, Manchester, UK), coupled with VISION software, at Centro de Materiais da Universidade do Porto (CEMUP), Porto, Portugal. The equipment used a monochromatic Al Kα X-ray source (1486.7 eV); operation conditions: 15 kV (90 W); Fixed Analyser Transmission (FAT) mode; 40 eV (regions ROI) and 80 eV (survey) of pass energy. C1s band at 284.6 eV was used as internal standard for binding energy (BE) calibration. CASAXPS software was selected for data analysis. The BET surface areas were obtained from the N_2_ adsorption isotherms collected on a Micromeritics ASAP 2060 gas sorption instrument (Hiden Isochema, Warrington, UK) at 77 K. Pre-outgassing of the samples at high vacuum at 130 °C for 24 h was undertaken to remove the physisorbed species from the surface of the examined samples.

### 3.3. Catalytic Studies

Typically, phenylbromide (0.5 mmol) and phenylboronic acid (0.6 mmol) as substrates, 2.5 mg composite as catalyst, potassium carbonate (1 mmol) as a base, and ethanol (2 mL) were introduced in a 250 mL stainless steel grinding jar and 10 stainless steel balls of 10 mm diameter were added. The grinding bowl was placed in the Retsch PM100 ball mill and the system was left under rotational speed 500 rpm, with rotational inversions every 5 min. At the end, 20 μL of the reaction mixture were taken and measured amount of DMSO-d_6_ was added and the sample was analyzed by ^1^HNMR (for more details see Supplementary Materials). The alkene additives (1,5-COD, 1-cycoctene, and cyclohexene) were taken in concentration 1 µL/2.5 mg.

## 4. Conclusions

In this study we demonstrated a simple and sustainable way to produce Pd(II) and Pt(II) and activated carbon heterogeneous nanocomposites. The preparation involves a simple, scalable, eco-friendly and fast ball milling technique and leads to dispersed catalytic materials, highly active for the Suzuki-Miyaura mechanochemical cross-coupling of bromobenzene with phenylboronic acid. The effect of very small amounts of olefins on the reaction yield was explored and in our most promising catalytic system (in the presence of AC-Pd2 and using cyclooctene as additive), relevant yields were achieved (*ca*. 80%). Moreover, the reported results show the importance of ball milling as a promising tool, in comparison with other energy inputs, in both synthesis and catalysis, and the advantages to combine a homogeneous catalyst with a supporting material under eco-friendly conditions. The green mechanochemical method will be further explored, expanding its scope and scaling up.

## Figures and Tables

**Table 1:** Denotation of the prepared composites.

	**Metal Precursor**	**Pd(OAc)_2_**		**K_2_PtCl_4_**	
**Support (100 mg)**		11 mg	22 mg	11 mg	22 mg
AC		AC-Pd1	AC-Pd2	AC-Pt1	AC-Pt2
MWCNTs		CNT-Pd1	CNT-Pd2	CNT-Pt1	CNT-Pt2
GO			GO-Pd2		

**Table 2:** Composition of the Pd/AC and Pt/AC composites.

Sample	Pd(wt%)		Pt(wt%)	
	Theoretical	Found (ICP-MS)	Theoretical	Found (ICP-MS)
AC-Pd1	4.7	4.13	-	-
AC-Pd2	8.5	7.63	-	-
AC-Pt1	-	-	4.6	4.47
AC-Pt2	-	-	8.5	7.28

**Table 3:** Brunauer—Emmett—Teller Surface Area Analysis (BET) characterization of Pd/AC and Pt/AC composites.

Sample	S_BET_ (m^2^/g)	Pore Volume (cm^3^/g)	Pore Size (nm)
AC	775	0.39	6.2
AC-Pd1	747	0.42	6.9
AC-Pd2	648	0.41	7.1
AC-Pt1	722	0.40	6.9
AC-Pt2	584	0.35	7.0

**Table 4:** Mechanochemical Suzuki—Miyaura cross-coupling reactions of bromobenzene with phenylboronic acid ^a^.

Entry	Catalyst	Solvent	Base	Time (min)	Yield (%) ^b^	TON^c^	TOF (h^−1^) ^d^
1	AC	EtOH	K_2_CO_3_	30	0	0	0
2	AC-Pd1	EtOH	K_2_CO_3_	10	27	139	835
3		EtOH	K_2_CO_3_	30	30	155	309
4		-	K_2_CO_3_	30	36	186	372
5	AC-Pd2	EtOH	K_2_CO_3_	10	41	114	683
6		EtOH	K_2_CO_3_	30	49	136	272
7		EtOH	K_2_CO_3_	180	69	192	64
8 ^e^		EtOH	Na_2_CO_3_	30	31	86	172
9 ^e^		EtOH	NaF	30	2	6	12
10 ^e^		EtOH	KF	30	34	94	189
11 ^e^		EtOH	Cs_2_CO_3_	30	35	97	194
12 ^f^		DCM	K_2_CO_3_	30	3	8	17
13 ^f^		ACN	K_2_CO_3_	30	5	14	28
14 ^f^		DMSO	K_2_CO_3_	30	4	11	22
15 ^f^		H_2_O	K_2_CO_3_	30	1.6	4	9
16	AC-Pt1	EtOH	K_2_CO_3_	10	26	228	1.4 × 10^3^
17		EtOH	K_2_CO_3_	30	5	44	88
18	AC-Pt2	EtOH	K_2_CO_3_	10	2.5	13	81
19		EtOH	K_2_CO_3_	30	2.3	12	25
20	GO-Pd2	EtOH	K_2_CO_3_	30	53	-	-
21	CNT-Pd1	EtOH	K_2_CO_3_	10	37	-	-
22	CNT-Pd1	EtOH	K_2_CO_3_	30	29	-	-
23	CNT-Pd2	EtOH	K_2_CO_3_	10	32	-	-
24	CNT-Pd2	EtOH	K_2_CO_3_	30	50	-	-
25	CaCO_3__5%Pd	EtOH	K_2_CO_3_	30	12	-	-
26	Pd(OAc)_2_	EtOH	K_2_CO_3_	30	75	34	68
27	K_2_PtCl_4_	EtOH	K_2_CO_3_	30	3	2.5	5

^a^ Reaction conditions: 2.5 mg catalyst, 0.6 mmol PhB(OH)_2_, 0.5 mmol PhBr, 1.0 mmol K_2_CO_3_, 2 mL EtOH; ball milling: 10 milling stainless still spheres of 10 mm diameter, 500 rpm with 5 min rotation interval. ^b^ NMR yield (%) based on the relative integration areas of the limiting reagent (PhBr) and the product (biphenyl). ^c^ TON = turnover number = number of moles of product per mol of metal content, found by ICP-MS, in the catalyst. An example of TON and TOF calculation is provided in ESM. ^d^ TOF = TON per hour. ^e^ 1.0 mmol base. ^f^ 2 mL solvent.

**Table 5:** Effect of different additives on the Suzuki-Miyaura cross-coupling reaction of bromobenzene with phenylboronic acid by AC-Pd2 ^a^.

Entry	Catalyst	Solvent	Base	Additive	Time (min)	Yield ^b^	TON ^c^	TOF (h^−1^) ^d^
1	AC-Pd2	EtOH	K_2_CO_3_	Cyclooctene	30	59	164	328
2		-		Cyclooctene	30	80	222	444
3		EtOH		Cyclooctene	180	38	106	35
4		EtOH		Cyclohexene	30	19	53	106
5		EtOH		1,5-COD	30	21	58	117
6		-		1,5-COD	30	39	108	217
7		-		EtOH	30	40	111	222
8 ^e^		-		NaCl	30	27	75	150

^a^ Reaction conditions: 2.5 mg catalyst, 0.6 mmol PhB(OH)_2_, 0.5 mmol PhBr, 1.0 mmol K_2_CO_3_, 2 mL EtOH; ball milling: 10 milling stainless still spheres of 10 mm diameter, 500 rpm with 5 min rotation interval. ^b^ NMR yield (%) based on the relative integration area of the limiting reagent (PhBr) and the product. ^c^ TON = turnover number = number of moles of product per mol of metal content, found by ICP-MS, in the catalyst. ^d^ TOF = TON per hour. ^e^ 0.6 g NaCl.

**Table 6:** Effect of the other energy inputs for the solid-state Suzuki-Miyaura cross-coupling reaction of bromobenzene with phenylboronic acid by AC-Pd2 ^a^.

Entry	Catalyst	Solvent	Base	Temperature (°C)	Time (min)	Yield (%) ^b^	TON ^c^	TOF (h^−1^) ^d^
CH								
1	AC-Pd2	EtOH	K_2_CO_3_	75	30	100	278	556
2					60	64	178	178
3					360	65	181	30
4				35	30	100	278	556
5					60	100	278	278
6					360	50	139	23
MW								
7	AC-Pd2	EtOH	K_2_CO_3_	75	30	61	169	339
8					60	100	278	278
9					360	69	192	32
10				35	30	52	144	289
11					60	56	156	156
12					360	47	131	22

^a^ Reaction conditions: 2.5 mg catalyst, 0.6 mmol PhB(OH)_2_, 0.5 mmol PhBr, 1.0 mmol K_2_CO_3_, 2 mL EtOH; ball milling: 10 milling stainless still spheres of 10 mm diameter, 500 rpm with 5 min rotation interval. ^b^ NMR yield (%) based on the relative integration area of the limiting reagent (PhBr) and the product. ^c^ TON = turnover number = number of moles of product per mol of metal content, found by ICP-MS, in the catalyst. ^d^ TOF = TON per hour. CH = conventional heating with oil bath; MW = under microwave irradiation (5–10 atm).

## Supplementary Materials

The following are available online at https://www.mdpi.com/1420-3049/25/12/2951/s1, Calculation of the theoretical content of metals in the composite materials, Figure S1: EDX analysis of the AC-Pd1 (a) and AC-Pd2 (b) composite materials; Figure S2: XPS survey spectra of the Pt/AC composites; Table S1. Surface atomic percentages determined by XPS for the pristine AC and within the studied Pd/AC and Pt/AC catalytic materials; Figure S3. XPS profiles of the studied Pd/AC and Pt/AC catalytic materials in C 1s and O 1s regions; Figures S4–S6: Selected ^1^HNMR showing yield calculation; Figure S7: N_2_ adsorption–desorption isotherms of the pristine AC; Figure S8: Effect of the different bases in the cross-coupling Suzuki–Miyaura reaction of bromobenzene and phenylboronic, catalyzed by AC-Pd2; Figure S9: Effect of the different solvents in the cross-coupling Suzuki–Miyaura reaction of bromobenzene and phenylboronic, catalyzed by AC-Pd2; Figure S10: Effect of the different supports.
