# Supplementary material for: Mechanochemical Preparation of Pd(II) and Pt(II) Composites with Carbonaceous Materials and Their Application in the Suzuki-Miyaura Reaction at Several Energy Inputs
Source: Molecules. 2020 Jun 26;25(12):2951. doi: 10.3390/molecules25122951 (PMC7355770; doi:10.3390/molecules25122951)
Supplement: Supplementary file 1 [file molecules-25-02951-s001.pdf]

Electronic Supplementary Material

for the paper

Mechanochemical Preparation of Pd(II) and Pt(II) Composites with Carbonaceous Materials and Their Application in the Suzuki-Miyaura Reaction at Several Energy Inputs

Mohamed M.A. Soliman [1,2], Andreia F. Peixoto [3], Ana P.C. Ribeiro [1], Maximilian N. Kopylovich [1,*], Elisabete C.B.A. Alegria [1,2,*] and Armando J. L. Pombeiro [1]

[1] Centro de Química Estrutural, Complexo I, Instituto Superior Técnico, Universidade de Lisboa, 1049-001 Lisboa, Portugal; mohamed.soliman@tecnico.ulisboa.pt (M.M.A.S.); apribeiro@tecnico.ulisboa.pt (A.P.C.R.); pombeiro@tecnico.ulisboa.pt (A.J.L.P.)

[2] Área Departamental de Engenharia Química, ISEL, Instituto Politécnico de Lisboa, 1959-007 Lisboa, Portugal

[3] REQUIMTE/LAQV, Departamento de Química e Bioquímica, Faculdade de Ciências, Universidade do Porto, Rua do Campo Alegre s/n, 4169-007 Porto, Portugal; andreia.peixoto@fc.up.pt

* Correspondence: maximilian.kopylovich@tecnico.ulisboa.pt (M.N.K.); ebastos@deq.isel.ipl.pt (E.C.B.A.A.); Tel.: +351 218317163 (E.C.B.A.A.)

Received: date; Accepted: date; Published: date

Academic editors: Raquel G. Soengas and Humberto Rodríguez-Solla

Calculation of the theoretical content of metals in the composite materials

AC-Pd1:

Atomic mass of Pd = 106.42 g/mol

Molar mass of $Pd(CH_3COO)_2$ = 224.51 g/mol

106.42 g/mol in 224.51 g/mol = x mg in 11 mg

x = 5.21 mg

5.21 mg of Pd in 111 mg of a composite = 4.7 %

AC-Pd2:

Atomic mass of Pd = 106.42 g/mol

Molar mass of $Pd(CH_3COO)_2$ = 224.51 g/mol

106.42 g/mol in 224.51 g/mol = x mg in 22 mg

x = 10.42 mg

10.42 mg of Pd in 122 mg of a composite = 8.5 %

AC-Pt1:

Atomic mass of Pt = 195.08 g/mol

Molar mass of K_2PtCl_4 = 415.09 g/mol

195.08 g/mol in 415.09 g/mol = x mg in 11 mg

x = 5.17 mg

5.17 mg of Pt in 111 mg of a composite = 4.6 %

AC-Pt2:

Atomic mass of Pt = 195.08 g/mol

Molar mass of K_2PtCl_4 = 415.09 g/mol

195.08 g/mol in 415.09 g/mol = x mg in 22 mg

x = 10.34 mg

10.34 mg of Pt in 122 mg of a composite = 8.5 %

EDX and XPS analysis

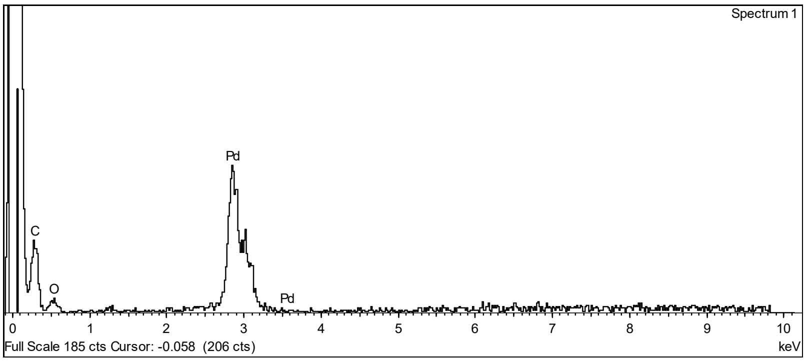

a)

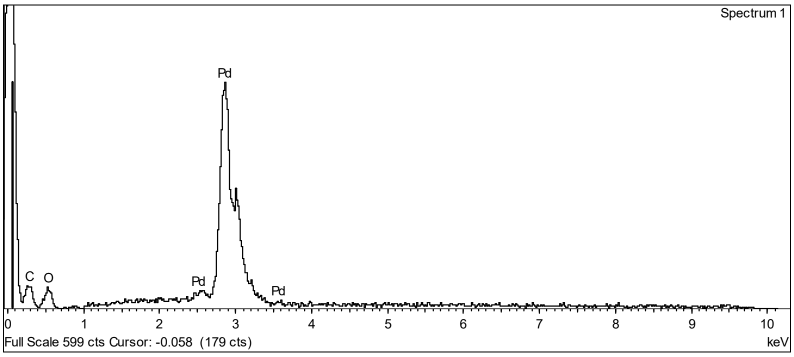

b)

Figure S1. EDX analysis of the AC-Pd1 (a) and AC-Pd2 (b) composite materials.

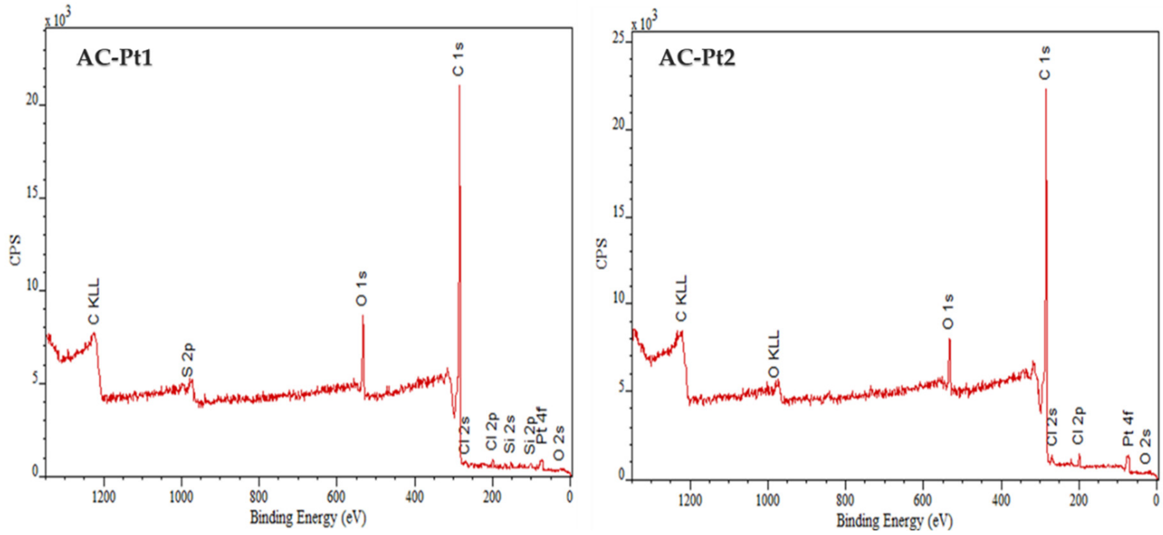

Figure S2. XPS survey spectra of the Pt/AC composites.

Table S1. Surface atomic percentages determined by XPS for the pristine AC and within the studied Pd/AC and Pt/AC catalytic materials.

**Material**	**Surface atomic %**						
	**Metal**	**C 1s**	**O 1s**	**$O_{\sim 531.5\ eV}$**	**$O_{533.3\ eV}$**	**$O_{534.2\ eV}$**	**O/C**
AC [2]	-	91.47	8.53	-	-	-	0.093
AC-Pd1[a]	0.82	90.06	9.12	6.92	-	2.33	0.101
AC-Pd2[a]	1.39	85.51	13.1	10.48	-	2.85	0.153
AC-Pt1	0.18	91.29	8.53	3.40	4.04	-	0.093
AC-Pt2	0.34	93.00	6.66	3.45	3.40	-	0.072

[a] In O 1s high resolution spectrum it was also observed a peak at 536.0 eV attributed to chemisorbed water.

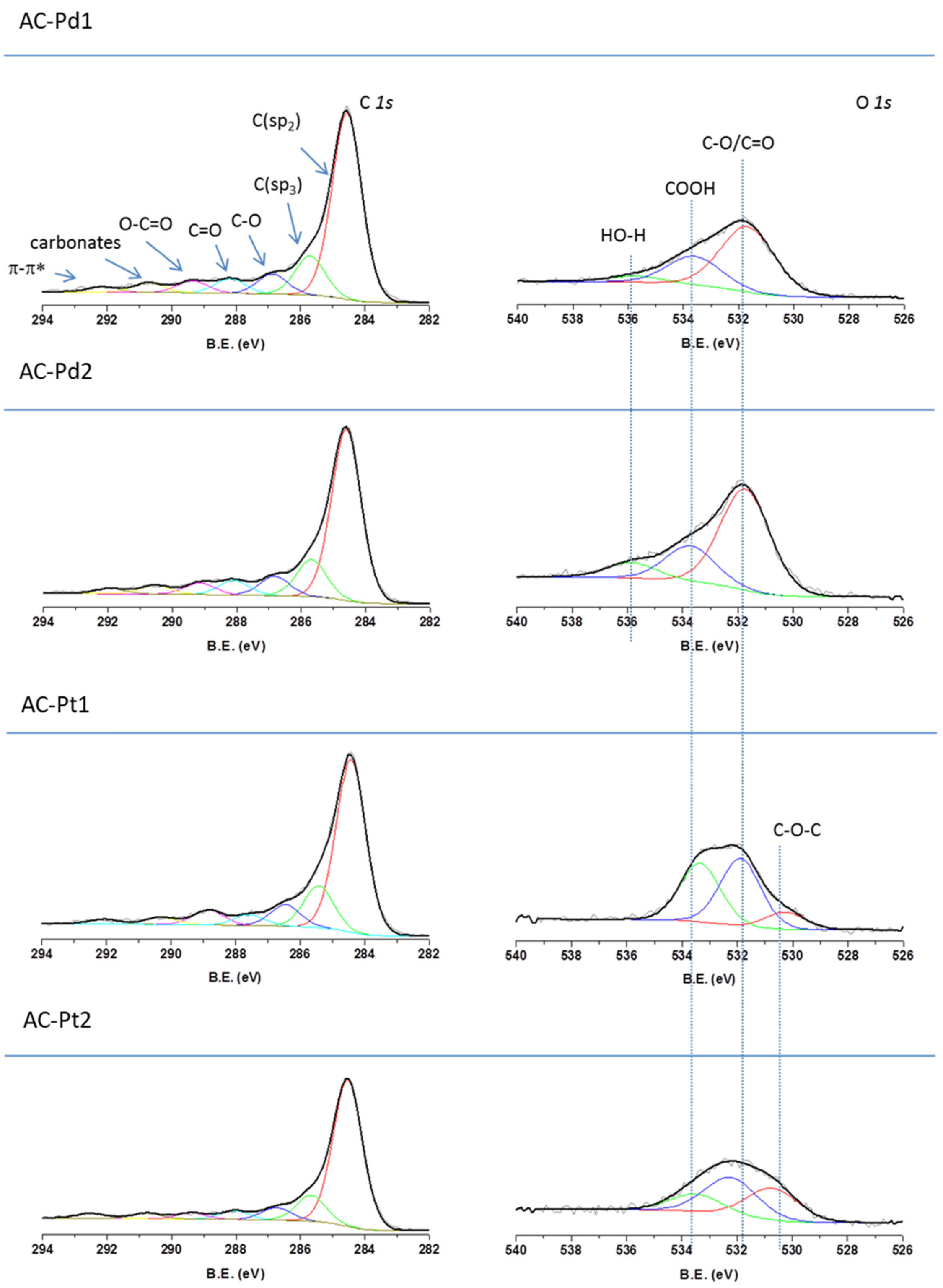

Figure S3. XPS profiles of the studied Pd/AC and Pt/AC catalytic materials in C 1s[1] and O 1s regions.

Catalytic studies

Calculation of yields by NMR

The yield was calculated by ^{1}H NMR based on the integration of well-defined selected peak areas of limiting reactant (bromobenzene) and the corresponding product (biphenyl).

Integral area of limiting reactant (PhBr) (x) → 2H

Integral area of product(biphenyl) (y) → 4H

Yield = [$\text{area}_{\text{product}}$ / ($\text{area}_{\text{substrate}}$ + $\text{area}_{\text{product}}$)] × 100

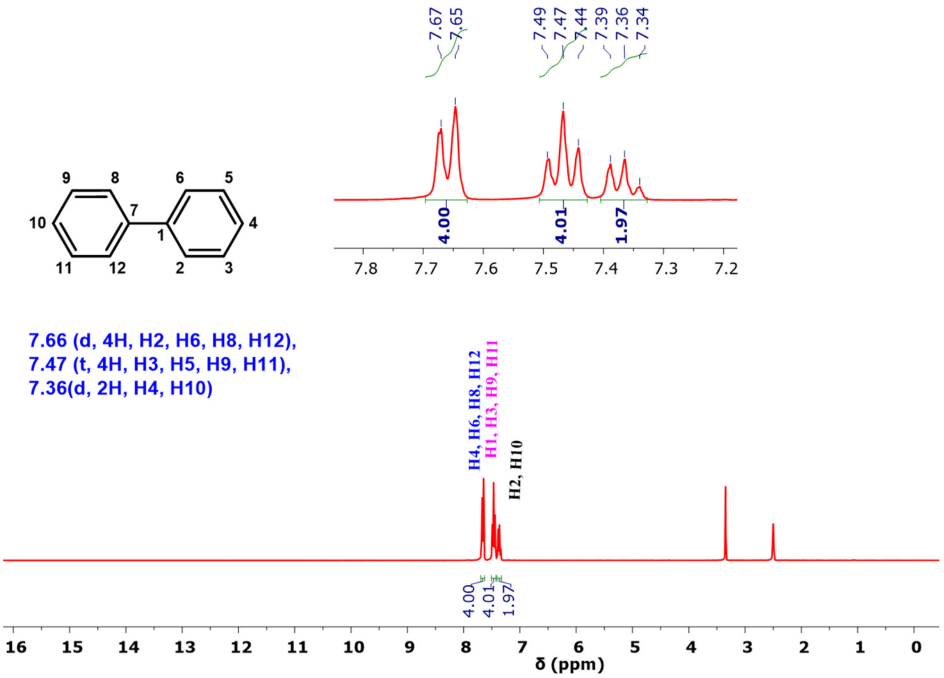

Figure S4. ^{1}H NMR of biphenyl, in DMSO-d_6.

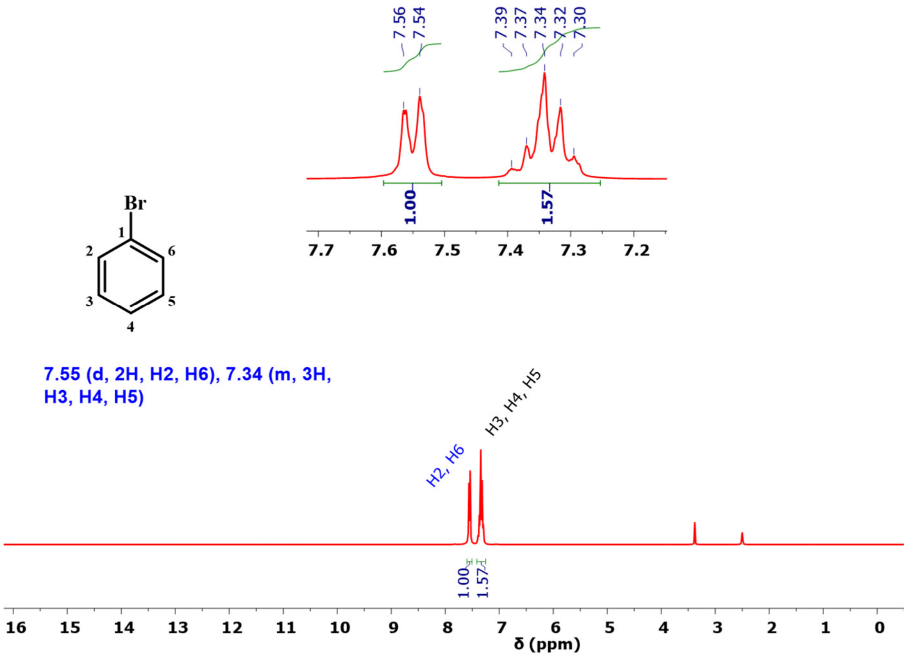

Figure S5. ^{1}H NMR of bromobenzene, in DMSO-d_6.

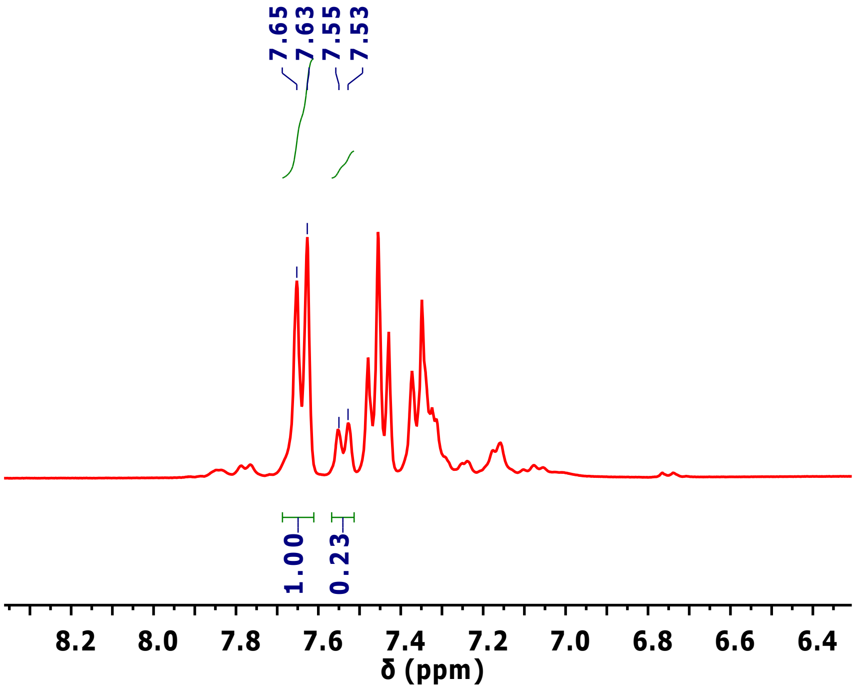

Figure S6. Selected ^{1}H NMR of Suzuki Miyaura reaction mixture the yield calculations (Yield 69%, Table 3, entry 7), in DMSO-d_6.

An example of calculation of TONs and TOFs

Table 4, entry 6 (Yield = 49%, reaction time = 30 min, 0.5 mmol PhBr (limiting reagent).

<u>AC-Pd1</u>:

Atomic mass of Pd = 106.42 g/mol

Found (ICP-MS) Pd content = 4.13wt%

2.5 mg of catalyst (composite) contains 4.13wt% or 0.10325 mg or 0.00097 mmol of Pd

TON = [(η/100)*0.5]/0.00097 = [(49/100)*0.5]/0.00097 = 136

TOF = 136/(0.5 h^{-1}) = 272 h^{-1}

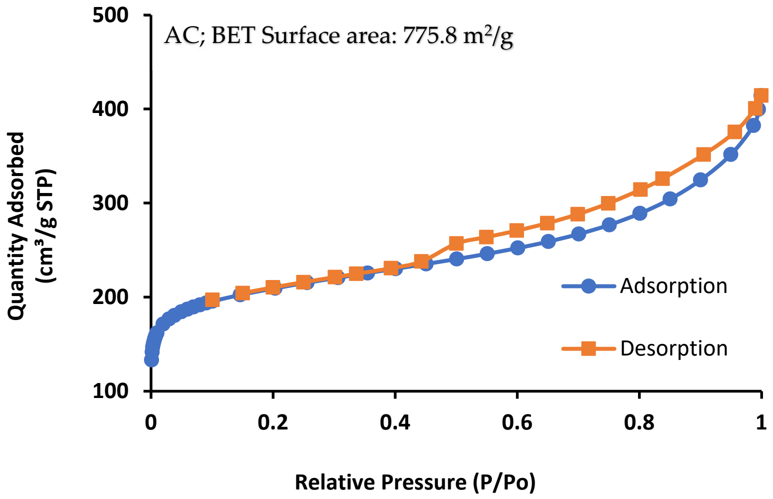

Figure S7. N_2 adsorption-desorption isotherms of the pristine activated carbon (AC).

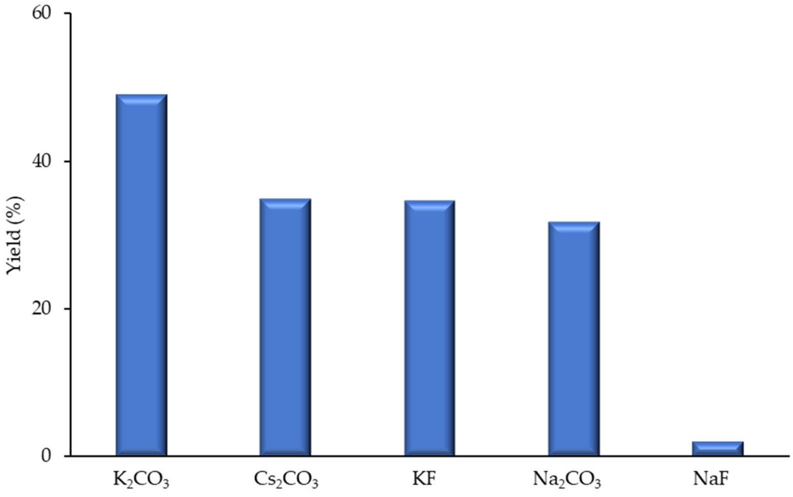

Figure S8. Effect of the different bases in the cross-coupling Suzuki-Miyaura reaction of bromobenzene and phenylboronic, catalyzed by AC-Pd2. Reaction conditions: 2.5 mg catalyst, 0.6 mmol $PhB(OH)_2$, 0.5 mmol PhBr, 1.0 mmol base, 2 mL EtOH, Ball mill: 10 milling spheres, 500 rpm, 500 rpm, rotation interval (5 min), 30 min.

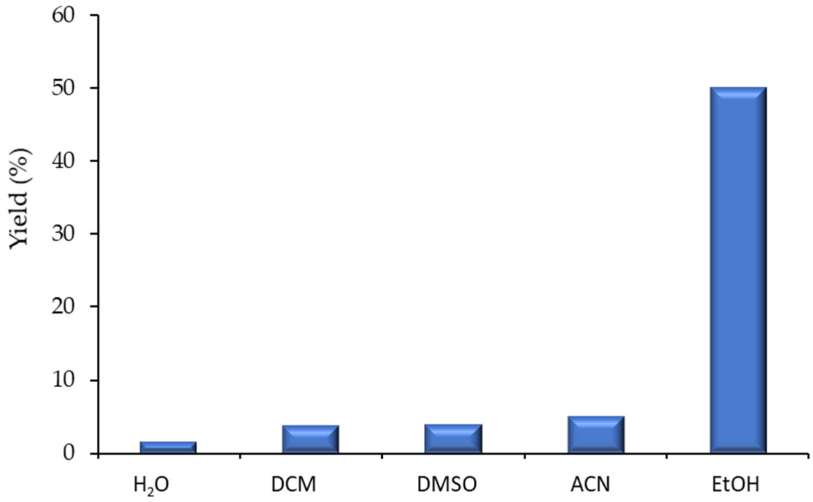

Figure S9. Effect of the different solvents in the cross-coupling Suzuki-Miyaura reaction of bromobenzene and phenylboronic, catalyzed by AC-Pd2. Reaction conditions: 2.5 mg of the composite, 0.6 mmol $PhB(OH)_2$, 0.5 mmol PhBr, 1.0 mmol K_2CO_3, 2 mL solvent, Ball milling: 10 spheres, 500 rpm, 30 min interval with 5 min inversion.

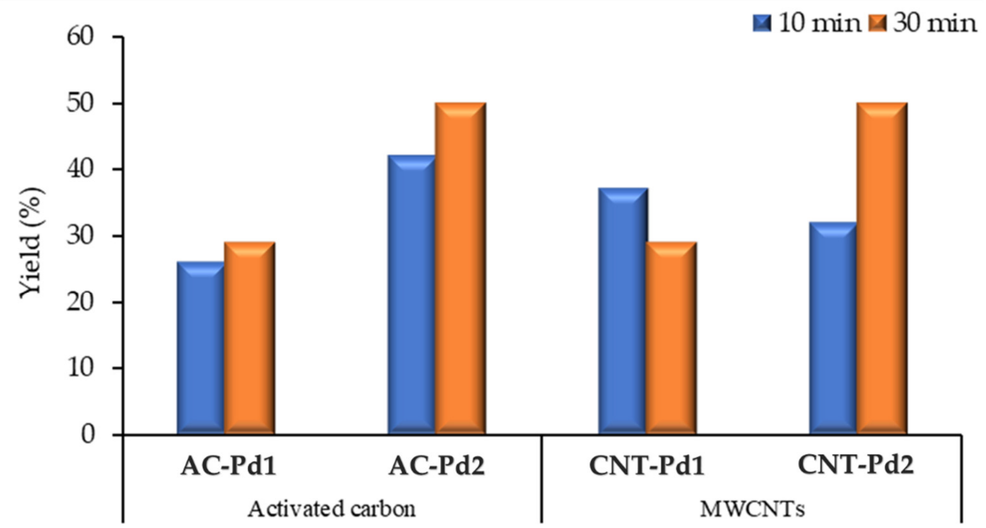

Figure S10. Effect of the different supports. Reaction conditions: 2.5 mg catalyst, 0.6 mmol $PhB(OH)_2$, 0.5 mmol PhBr, 1.0 mmol base, 2 mL EtOH. Ball milling: 10 spheres, 500 rpm, 30 min interval with 5 min inversion.

References

[1] Y.-C. Chiang, Y.-J. Chen and C.-Y. Wu, *Materials* **2017**, *10*, 1296.

[2] Shuttleworth, P. S.; Baccile, N.; White, R.J.; Nectoux E.; Budarin V.L. Bulk and surface analysis of carbonaceous materials, in *RSC Green Chemistry Series No.32: Porous carbon materials from sustainable precursors*, R.J. White (ed.). RSC Publisher, **2015**, p. 311-354.
